# PW02-012 - First clinical description of an infant with DITRA

**DOI:** 10.1186/1546-0096-11-S1-A152

**Published:** 2013-11-08

**Authors:** L Rossi-Semerano, M Piram, C Chiaverini, D De Ricaud, A Smahi, I Koné-Paut

**Affiliations:** 1 Department of Paediatrics and Paediatric Rheumatology, Bicêtre Hospital, National Reference Centre for Auto-inflammatory Diseases, Le Kremlin-Bicêtre, France; 2 Service de dermatologie et centre de référence des épidermolyses bulleuses héréditaires Hôpital Archet 2 CHU de Nice, France; 3 Service de pédiatrie, GCS CHU-Lenval, Nice, France; 4 U781 INSERM, Hôpital Necker Enfants Malades, Paris, France; 5 Department of Paediatrics and Paediatric Rheumatology, Bicêtre Hospital, National Reference Centre for Auto-inflammatory Diseases, Le Kremlin Bicêtre, France

## Introduction

Interleukin-36-receptor antagonist deficiency (DITRA) is a recently described auto-inflammatory disease^1^, characterized by repeated flares of generalized pustular psoriasis, high fever, asthenia and systemic inflammation. This condition is caused by homozygous missense mutation in the *IL36RN* gene, encoding the interleukin-36-receptor antagonist (IL-36Ra), an anti-inflammatory cytokine. We report herein the first exhaustive clinical description of an infant with DITRA, who was successfully treated with anakinra.

## Case report

Y.M. is the first son of Tunisian consanguineous parents who developed, at two weeks of life, an erythematous and scaly eruption, with subsequent rapid evolution toward generalized pustular psoriasis. Afterwards, cutaneous flares of diffuse erythematous rash and pustules involving the whole body appeared, with a once weekly periodicity. Intense irritability was present during flares without fever. Moreover, since 1 month of age the infant presented diarrhea, dysphagia and reduced feeding rate, with failure to thrive. Laboratory tests during acute flares showed marked leukocytosis, thrombocytosis and anemia without C-reactive protein elevation. Skin biopsy and clinical presentation were consistent with pustular psoriasis, nevertheless, the patient did not respond to high-potency topical corticosteroids and retinoid acid.

As the patient presented repeated skin flares early after birth, as well as serious constitutional distress with failure to thrive, an auto-inflammatory syndrome like DIRA (interleukine-1 receptor antagonist deficiency)[2] or DITRA was considered. The hypothesis was reinforced by parental consanguinity, and absence of skin lesions improvement under standard topical treatment. Genetic analyses showed a homozygous mutation in the *IL36RN* gene (L27P) which represents the same mutation recently described in DITRA patients[1,3]. At 6 months we started treatment with the recombinant IL-1 receptor antagonist anakinra with efficacy both on constitutional symptoms and skin involvement.

## Discussion

To the best of our knowledge, we report the first detailed clinical description of an infant with DITRA. Even if neonatal onset has been already reported[1], no detailed clinical description was provided, probably due to late diagnosis. Our clinical report brings new clinical characteristics and educational iconography.We even report, for the first time, a favorable clinical response of this disease to anakinra treatment.

## Disclosure of interest

L. Rossi-Semerano: None Declared, M. Piram: None Declared, C. Chiaverini: None Declared, D. De Ricaud: None Declared, A. Smahi: None Declared, I. Koné-Paut Grant / Research Support from: Educational and research grant from Swedish Orphan Biovitrum, Consultant for: Consultant fee from Novartis
